# Social responses of travelling finless porpoises to boat traffic risk in Misumi West Port, Ariake Sound, Japan

**DOI:** 10.1371/journal.pone.0208754

**Published:** 2019-01-02

**Authors:** Naruki Morimura, Yusuke Mori

**Affiliations:** Kumamoto Sanctuary, Wildlife Research Center of Kyoto University, Uki, Kumamoto, Japan; University of Minnesota, UNITED STATES

## Abstract

Anthropogenic effects have created various risks for wild animals. Boat traffic is one of the most fatal risks for marine mammals. Individual behavioral responses of cetaceans, including diving behavior such as changing swimming direction and lengthing inter-breath interval, to passing boats is relatively well known; however, the social function of cetacean responses to boat traffic in a natural setting remains poorly understood. We focused on describing the behavioral responses of single and aggregated finless porpoises to boats passing at Misumi West Port, Ariake Sound, Japan, by using a drone characterized with a high-precision bird’s-eye angle. During the study period, we collected 25 episodes of finless porpoise responses to boats passing by. A mean (± SEM) of 5.1 ± 1.0 individuals were observed for each episode. The primary response to passing boats was avoidance by dive, which implies boat traffic is a substantial disturbance to finless porpoises that travel along the seawater surface daily. The diving duration decreased significantly with an increase in the number of aggregated individuals. The diving and floating reaction times were 10.9 ± 2.3 s and 18.7 ± 5.0 s, respectively. There was no significant difference between the reaction times indicating that each individual was motivated to keep the group cohesion consistent when floating even after the risk had dissolved, which is comparable to the behavior of porpoises that dive when riskier conditions are present, such as when a boat approaches an aggregation. Our findings provide new insights on the sociality of finless porpoises even though there were limitations, like an inability to identify a specific individual. The drone enabled us to observe the social behavior of finless porpoises and other cetaceans at an unprecedented resolution, which may lead to a better understanding of the evolutionary diversity of intelligence and sociality and the bridge to human evolution.

## Introduction

Anthropogenic impacts have created a variety of risks for non-human animals (hereafter “animals”) in the wild [1, 2, 3]. Traffic accidents are one of the major causes of death for both terrestrial and marine mammals inhabiting areas close to anthropogenic activities. Roadkill can lead to wildlife mortality that exceeds natural rates [4]. In 2002, 31.1% of all known manatee (*Trichechus manatus latirostris*) deaths in Florida were caused by boat strikes [5]. Under non-fatal risks, wild animals are required to adapt to dangerous situations. Wild chimpanzees (*Pan troglodytes*) in Bossou, Guinea, showed a tendency to wait longer to cross larger roads than smaller roads [6]. The dugong (*Dugong dugon*) reduces its feeding time budget to 0.8–6% when boats passed within 50 m [7]. The behavioral responses of cetaceans to boats are relatively well known in terms of individual movement and diving behavior. Cetaceans respond directly to boats by changing their swimming direction [8, 9, 10], lengthening their inter-breath intervals [11, 12], and reducing their inter-individual distances [13].

In contrast, few studies have quantified the social aspects of the behavioral responses to risks. Long-finned pilot whales (*Globicephala melas*), which are social toothed whales that form stable matrilineal groups, formed larger group sizes to increase their social cohesion during experimental disturbances of killer whale sound playbacks, tagging efforts, and naval sonar exposure [14]. Bottlenose dolphins (*Tursiops aduncus*) responded to the presence of commercial dolphin watch/swim boats by increasing their whistle rates, which may affect group cohesion [15]. Nonetheless, the social function of cetacean responses to different disturbances and external risks in a natural setting remains poorly understood, even though most cetacean groups display some form of social structure which that varies from ephemeral aggregations to long-lasting social bonds within the groups [16].

Innovations in research technology may enable advanced approaches for studying the social aspects of cetacean behavior. Unmanned aerial vehicles (UAVs), or drones, are rapidly becoming a more common data-gathering tool for wildlife research and monitoring [17, 18]. UAVs can potentially collect ecological and behavioral data at unprecedented spatial and temporal scales across various topographic regions. Ref. [19] showed that a UAV could detect up to 52.4% more camouflaged or invisible Iceland gull (*Larus glaucoides*) chicks than that of ground observers during a population census. UAVs can produce a sea turtle length scale with accuracy within centimetres, which is far beyond the data quality of typical satellite-derived or manned-aircraft images [20]. Ref. [21] used a drone to estimate the blow (exhaled breath condensate) of the blue whale (*Balaenoptera musculus*). Biologists, therefore, have quickly come to appreciate that drones can offer data-gathering opportunities that are otherwise impossible or extremely expensive with traditional methods [17, 22, 23, 24].

The finless porpoise (*Neophocaena asiaeorientalis*) is a small species that has no dorsal fin, which reduces their visibility for surface observations; therefore, the sociality of this species in the wild is largely unknown. Ref. [25] showed that stable social bonds form, at least, between mothers and calves. Using a time-synchronized bio-logging system, a recent study on the Yangtze finless porpoise (*N*. *a*. *asiaeorientalis*) showed an adult male engaged in synchronized diving with an immature-female and a juvenile-male, suggesting multiple forms of social associations that are non-opportunistic aggregations [26]. At the Misumi West Port, Ariake Sound, Japan, settled finless porpoises (*N*. *a*. *sunameri*) are well known among the local people, while research and/or touristic observations are not common because of the reduced underwater visibility. Our preliminary drone survey was conducted in early 2017 and showed the potential for routine direct observations of the finless porpoise. The historical Misumi West Port is active with boat traffic for fisheries and small-scale material transport. Finless porpoises occasionally encounter boats during their travel in the bay. There is only one anecdotal account of a finless porpoise boat strike during the last decade (Japan Fisheries Cooperatives in Misumimachi, unpublished data). Ref. [27] calculated that 9% of finless porpoise mortality could be attributed to boat strikes in Hong Kong, even though the records of finless porpoise fatalities from boat traffic have been mostly indirect or anecdotal.

Therefore, in this present study we focused on describing the behavioral responses of both single and aggregated finless porpoises to passing boats, which can pose a fatal risk, using a high-precision drone with an angled bird's-eye view. We assumed, under the premise of previous studies, that finless porpoises have developed relatively ephemeral social relationships that are simply structured [25, 28], since > 100 individuals show occasional aggregations only [29]. Therefore, the null hypothesis was that there were no social effects on the behavioral responses to passing boats; namely, there would be no difference in diving behavior regardless of the number of aggregated individuals. Some asocial animals, like female brown bears (*Ursus arctos*) with yearling cubs, have a tendency to disperse from aggregations, even when food resources are abundant, as a conflict avoidance strategy with conspecifics [30]. In contrast, social animals have evolved species-specific affiliative behaviors for mitigating the impacts of a risky event among the group members. For example, wild spider monkeys (*Ateles geoffroyi*) use embracing to mitigate conflict dynamics within their groups [31]. Rubbing against another individual is one of the most common affiliative behaviors in the bottlenose dolphin and other cetaceans [32, 33]. Thus, proximity, gathering, and affiliative interactions during surface swimming both before and after the passing of the boats can be behavioral clues that represent the social relationships between finless porpoises. Again, the null hypothesis was that there would be no tendency for social cohesion and affiliative interactions when boats passed.

## Methods

### Study site

**Fig 1:**
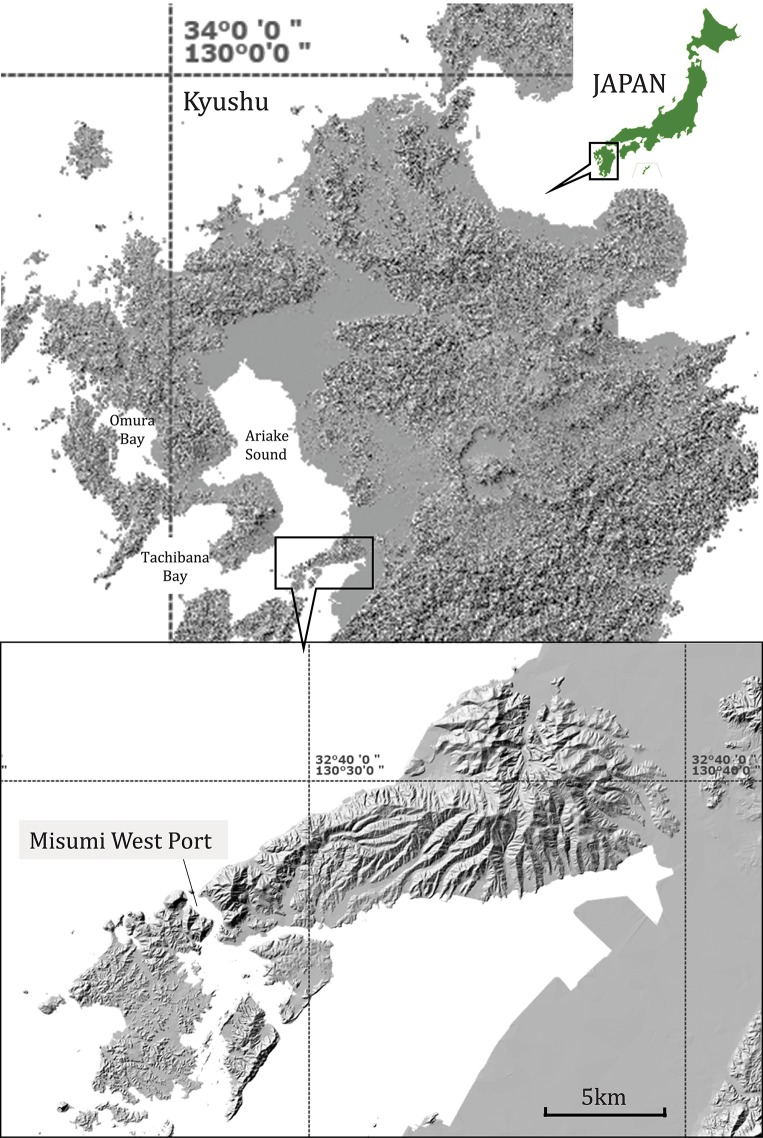
Study site of Misumi West Port in Ariake Sound, Japan. This map is based on the Digital Map (Basic Geospatial Information) published by the Geospatial Information Authority of Japan.

*Neophocaena asiaeorientalis sunameri* is distributed throughout the shallow (usually <50 m deep) coastal waters of Japan [34]. Fieldwork was conducted in an area of 1.2 km^2^ off the Japanese coast in the marine waters of the Misumi West Port (Fig 1; 32° 37' 7.4" N, 130° 27' 13.5" E) between March 2017 and May 2018. During the study, we regularly launched a drone to conduct observational studies on finless porpoises in the bay. Our year-round observations showed single and aggregated finless porpoises, sometimes exceeding more than 60 individuals, engaging in a variety of behavioral repertoires including solitary and group feeding, travelling, copulation, and mother-calf interactions. We conducted the observations without identifying each individual. Water visibility during the study period was usually less than 2 m, but varied daily depending on the weather and tide conditions.

### Data collection

#### Drone observations

Surface behavior of finless porpoises was recorded using drones (DJI Co. Ltd, Mavic Pro). The aircrafts were manually controlled with the Litchi application (ver. 4.6.1-g) for Android OS (VC Technology Ltd). We collected video footage that totalled 15 min for a single flight. The flight was conducted from 5 pm until sunset for a maximum of 3 h per day. The flight height of the drone varied from 40 to 149 m above sea level (asl) and was adjusted to provide the most preferable recording conditions for targeting individuals. Thus, the presence and noise of the aircraft had to have limited impact on the animal’s surface behavior [35], while its distance potentially influenced its ability to determine the animal’s social interactions in detail. The pitch angle value of the camera embedded in the drone was kept at -90 degrees during the flight, but the pitch angle varied if necessary to record a whole episode continuously. The observation was repeated daily, but was cancelled if the weather conditions were unsuitable.

#### Boat passing episodes

A drone was used to observe when a boat approached the finless porpoises; the drone was 149 m above the targeted animals with -90 degrees of pitch angle. A single episode of a boat passing consisted of four continuous scenes: 1) an initial scene of finless porpoises swimming at the water’s surface, 2) a scene of a boat approaching the targeted animals, 3) a scene of the animals’ response to the passing boat, and 4) a final scene of the targeted animals swimming again at the water’s surface. The order of these scenes could have varied among episodes, such that a porpoise may have started diving before a boat appeared within the drone’s camera view. Episodes lacking any of the four scenes were omitted from further analysis. Thus, each episode was collected using a focal group sampling method.

### Data analysis

#### Aggregation and the classification of solitary and social episodes

We quantified the surface behavioral data by reviewing the video footage; however, no baseline for social behavior in finless porpoises was available because the sociality is mostly unknown owing to a limitation in previous observation opportunities. We defined an aggregated group, with reference to a previous study on bottlenose dolphins [10, 36], as all the porpoises that were moving in the same direction and often engaged in the same activity located within the drone’s camera view, which had an approximate area of 130 m long by 190 m wide at 150 m asl with -90 degrees pitch value; Not all porpoises in the camera view were included in the aggregate group if the porpoises were substantially distanced from the group with the passing of the boats or if they were engaged in a different behavior. An aggregation did not guarantee that porpoises within a group were associated with each other. Based on the number of individuals in an aggregation, we classified the sociality of each episode as solitary or social and determined the group size.

#### Diving episodes and classifying boat avoidance

Diving behavior is well known as a common cetacean response to passing boats [12, 14, 26]. Behavioral risk avoidance is responsible for taking time and energy away from other critical behaviors like survival and reproduction. Thus, we classified the response of each episode as avoidance or non-avoidance. Moreover, in order to evaluate aggregated group cohesion, the reaction times of diving and floating were calculated, which represented the amount of time between the initial phase of the first engaged individual and the final phase with the last engaged individual. The diving duration was determined by calculating the amount of time between the final phase of diving and the initial phase of floating.

#### Social interactions: Gathering and rubbing behaviors

Group cohesion characterizes the social relationships among a group of social animals [37]. Affiliative behavior, reconciliation, and consolation, which has been shown to mitigate the stressful impact from a previous negative experience in primates and other social animals [38, 39], are expressed by the proximity between individuals, while aggressive interactions may occur among conspecifics [40]. In order to evaluate the tendency of social cohesion in finless porpoises in response to the passing of boats, the number of aggregated individuals was compared between the sequences before and after the passing of boats by categorizing each episode as a dispersal (a decrease in the group number after the passing), equivalent (no change), or gathering (an increase in the group number after the passing). If porpoises are solitary, a dispersal tendency with weak social cohesion is expected more than an increase of individuals gathering.

In contrast, finless porpoises can engage in affiliative behavior when boats pass, if they have relatively higher group cohesion, which suggests the presence of social bonds among them. Rubbing behavior is one of the most common affiliative behaviors in finless porpoises and other cetaceans [32]. In this study, rubbing behavior was defined as one porpoise rubbing its body on another individuals body. Additionally, we determined rubbing behavior only under the limited conditions of parallel surface swimming of adult dyads without a flight reaction from the rubbing. Since the distance between the targeted porpoises and the aircraft was potentially more than 150 m, there may have been less precision for comparing these observations with the direct observations made in previous studies (e.g., [41]). We classified each episode as rubbing or non-rubbing based on the presence or absence of the rubbing behavior. For a before sequence of a boat passing we reviewed each 30-sec video before the first individual dived. For an after sequence, we reviewed each 30-sec video footage after the first individual floated. We compared the average number of aggregated individuals between rubbing and non-rubbing episodes with the combined before and after boat passing sequences to evaluate if there was a group size influence on the rubbing behavior of the finless porpoises.

#### Influence of the size of and distance from the boats

The characteristics of the boat approaching the porpoises might be a factor directly influencing the response of the finless porpoises. We roughly estimated the boat size and distance between the boat and porpoise on the basis of the video footage recorded by the drone. The boat size referred to a boat's length. The distance between a boat and a porpoise was determined by measuring the minimum distance between the boat and the porpoise, which was the first individual that dived during the diving episodes or the individual nearest to a boat in the non-diving episodes. The object size in a still image was calculated on the basis of the following specifications of the lens 28 mm focal length in 35 mm format equivalent (36 x 24 mm sensor size). Under these conditions, a known 6.7 m-length object (a boat) was estimated as 6.7 m at 40 m height, 6.4 m at 70 m asl, 6.3 m at 120 m asl, and 6.3 m at 150 m asl, respectively. We did not include a correction to account for the distortion of lens equipped with the drone. Because these measures were a rough estimation, the boat size and the distance were reported as integers.

### Statistical analysis

We examined whether the number of aggregated individuals during a boat pass was correlated, using Pearson's product-moment correlation, with the two behavioral aspects of diving: the reaction times of diving and floating. As a direct social response to boats passing, the correlation between diving duration and the number of aggregated individuals was tested. We also tested the correlation of diving duration with boat size and the distance from the boat in order to examine the influences of boat characteristics on porpoises’ response towards boats. Additionally, to characterize the social cohesion tendency for the reaction times of diving and floating, the average lengths of diving and floating reaction times were compared using a Welch two sample t-test.

If the finless porpoises had a tendency to mitigate the risks of a boat passing through their aggregation, social interactions among aggregated individuals could be influenced by the group size. Thus, the average number of aggregated individuals was compared with the gathering responses (gathering vs. non-gathering episodes) and the rubbing behavior (rubbing vs. non-rubbing episodes) using a Welch two sample t-test. Moreover, if finless porpoises had weak social relationships, the combined data of gathering and rubbing behaviors may expose their sociality. Therefore, social and asocial episodes that represented the presence or absence of the rubbing behavior or the gathering before and after the boats passed was compared with the average number of aggregated individuals.

Statistical tests were performed using R statistical software, version 3.5.0 [42]. We considered values of P < 0.05 as statistically significant.

### Ethics statement

All research activities in the sea area of the Misumi West Port were carried out under the permits issued by Uki City Government, Kumamoto, Japan. Kumamoto Coast Guard supported our research, especially on the security of boat traffic control and the related human activity. Drone flight, including the operation without a visual line of sight, was approved by the Osaka Regional Civil Aviation Bureau (permit no. 1009/no. 441). The research protocol was approved by the institutional committee of the Wildlife Research Center, Kyoto University (permit no. WRC-2017-001A and WRC-2018-002A).

## Results

**Fig 2:**
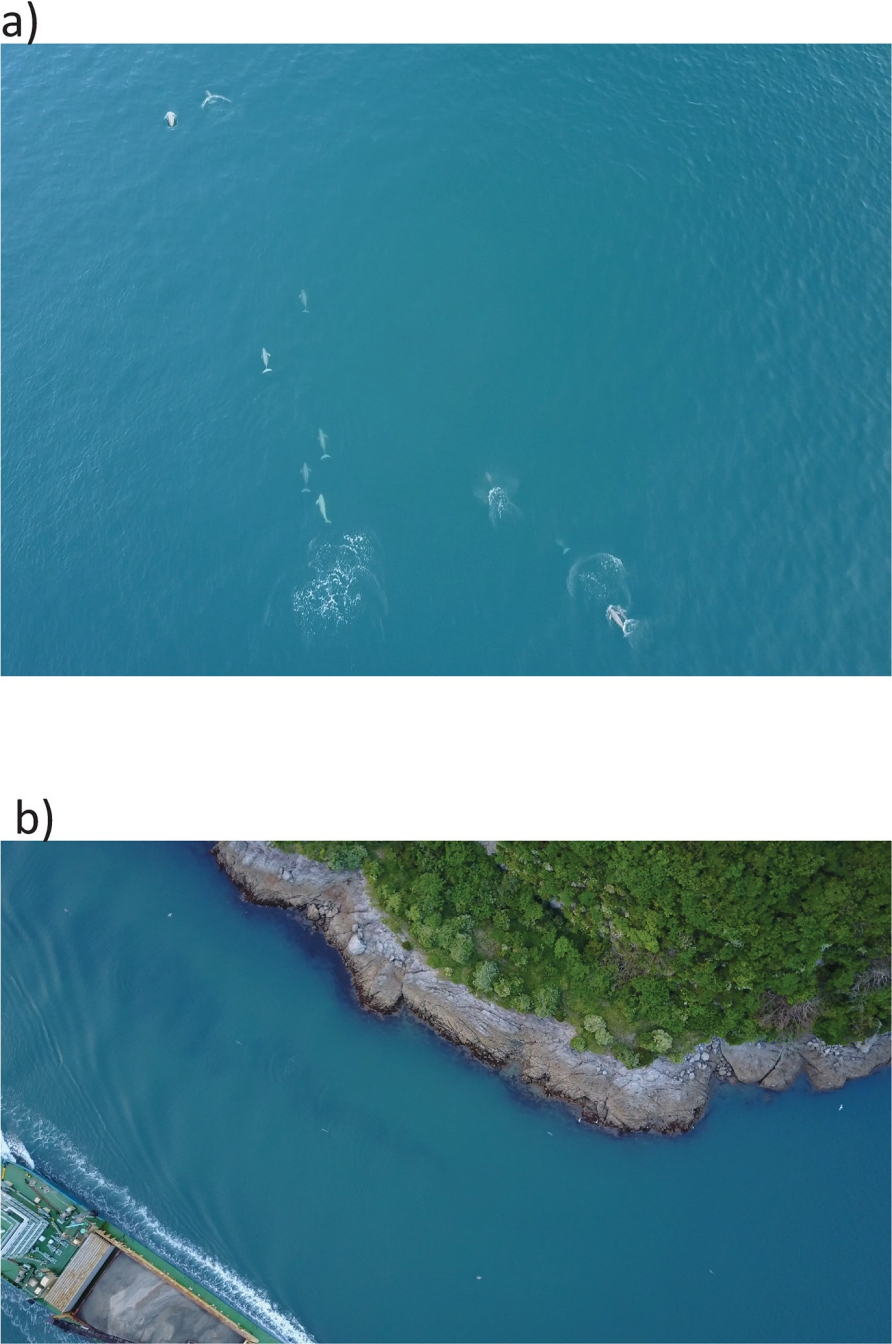
a) Aggregation of 11 finless porpoises swimming at the seawater surface during episode 1; b) Five finless porpoise individuals swimming near a vessel and three seabirds flying in non-avoidance during episode 4.

**Fig 3:**
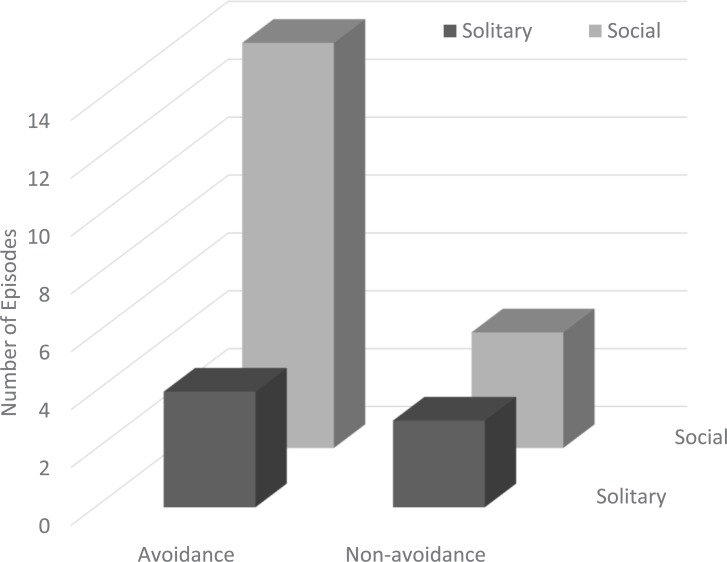
Comparison between the number of episodes of the avoidance response to boat passes and overall sociality.

In total, 25 episodes of finless porpoises’ responses to boat passing were collected during the study period (Fig 2). Of these episodes, 18 (72.0%) were on porpoise aggregations that ranged from 2 to 21 individuals (Table 1). A mean (± SEM) of 5.1 ± 1.0 individuals was observed in each episode. The average aggregation size was 6.7 ± 1.2 individuals. The average estimated boat size was 20 ± 4.5 m; however, we failed to film the boat but did capture the wake at the passing near a porpoise in episode 15. The estimated average distance between a boat and a porpoise that was the first individual to dive during the diving episodes or the one nearest to the boat in the non-diving episodes was 55 ± 6.2 m. The most common response to the passing of boats was avoidance, which implies that the boats passing disturbed the finless porpoises that travelled at the seawater surface. All the targeted porpoises dived in 18 episodes (Fig 3). However, all or some of the porpoises did not avoid the boats by diving in episodes five and two, respectively. A solitary porpoise in three episodes kept swimming close to a passing boat at the seawaters surface.

**Table 1:** Estimated boat size and distance from porpoise(s) based on drone images.

Episode	Aggregation size (#individuals)	Boat size (m)	Distance from boat (m)
#01	11	10	>12
#02	8	10	19
#03	21	10	>35
#04	6	>73^a^	43
#05	13	8	41
#06	4	15	>81
#07	10	12	>69
#08	1	54	>64
#09	1	8	65
#10	1	15	113
#11	3	16	>15
#12	3	7	>115
#13	1	10	>61
#14	1	70	58
#15	1	Unknown^a^	>42^b^
#16	6	11	>80
#17	2	14	49
#18	2	9	33
#19	8	7	51
#20	2	9	>31
#21	1	10	>40
#22	6	8	>26
#23	2	75	>33
#24	5	10	>133
#25	9	14	>60

Note

a: No image or partial image of the boats were recorded by a drone at the passing of porpoise(s).

b: The estimation was a measure of distance between porpoise and the wake that the boat created.

The number of aggregated individuals increased after a boat passed, namely a social response of gathering, in six episodes, but the number of individuals was the same before and after boats passed in nine of the 18 aggregated episodes. A disperse response, where the number of individuals decreased after a boat passed, only occurred in three episodes. Thus, finless porpoises maintained or enhanced their aggregation in 83.3% of all the aggregation episodes.

**Fig 4:**
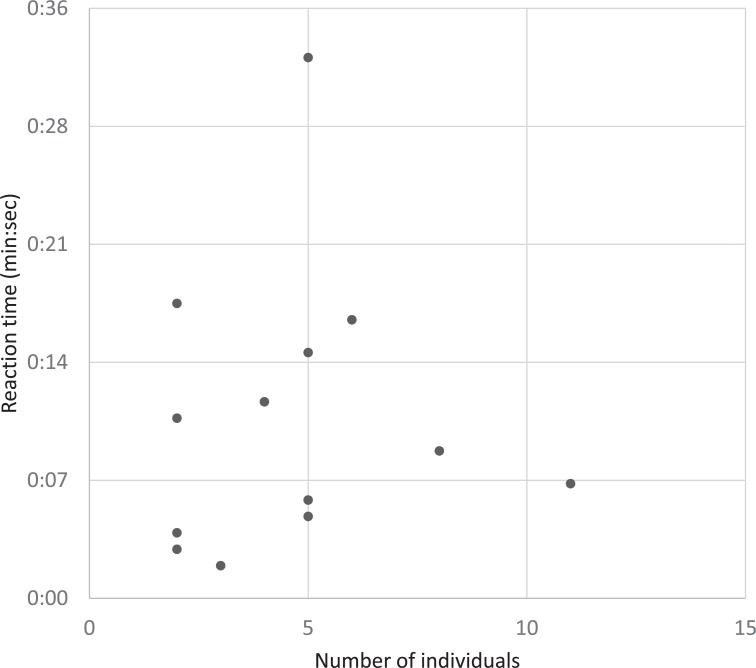
The correlation between reaction time of diving and group size during 13 group avoidance episodes.

**Fig 5:**
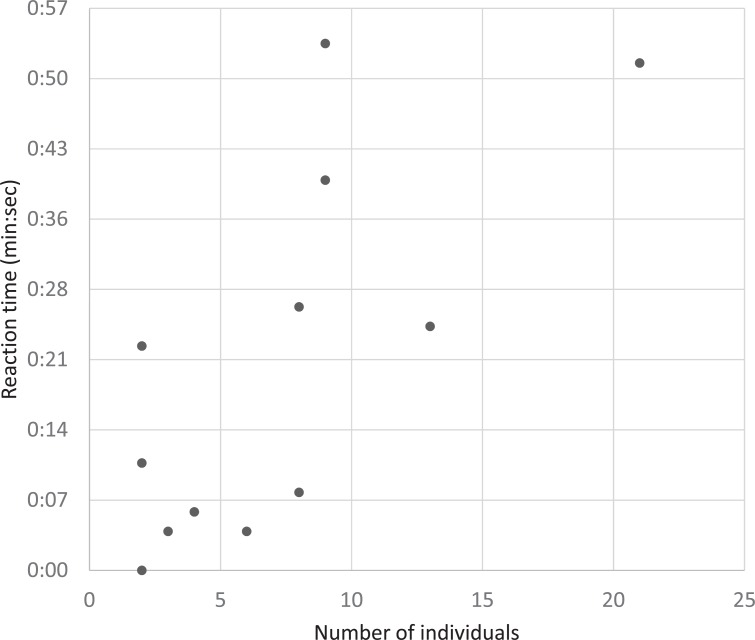
The correlation between reaction time of floating and group size during 14 group avoidance episodes.

Of the 18 avoidance episodes, 14 episodes were with aggregated individuals. The reaction time of diving when boats approached was significantly correlated with the number of aggregated individuals (*r* = 0.67, *t*_12_ = 3.15, *P*-value < 0.01). However, when a single reaction time outlier (46 sec. of diving reaction time by 21 individuals in episode 3) was removed from the analysis, the correlation among the 13 episodes was not significant (*r* = 0.06, *t*_11_ = 0.20, *P*-value = 0.84; Fig 4). In episode 3, 13 individuals dived first and eight individuals followed and 21 individuals gathered after the boat passed. In contrast, the reaction time of floating during 14 episodes was significantly correlated with the number of aggregated individuals (*r* = 0.74, *t*_12_ = 3.79, *P*-value < 0.01; Fig 5). Even though the aggregation effect on the reaction times were different between the diving and floating phases, the mean of those reaction times were not significantly different from each other (10.9 ± 2.3 sec. in diving vs. 18.7 ± 5.0 sec. in floating; *t* = -1.42, *df* = 18.47, *P*-value = 0.17). Thus, aggregated porpoises responded to boats passing similarly with the diving and floating reactions.

**Fig 6:**
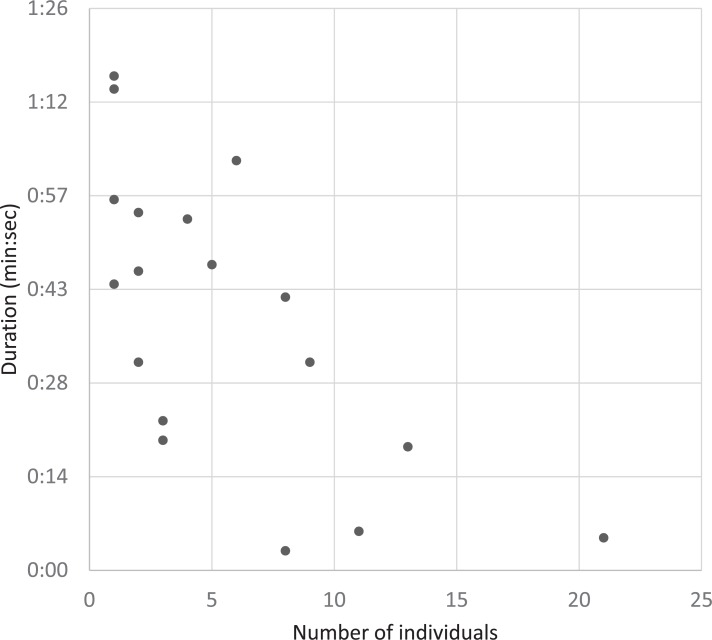
Correlation between diving duration and group size.

Moreover, the diving duration among 18 avoidance episodes significantly decreased with the number of aggregated individuals (*r* = 0.74, *t*_12_ = 3.79, *P*-value < 0.01). The more finless porpoises that aggregated when boats passed the more the diving duration decreased (Fig 6). Additionally, the diving duration did not correlate with boat size or the distance between porpoise(s) and boat (boat size: *r* = 0.08, *t*_16_ = 0.31, *P*-value = 0.75; boat distance: *r* = 0.35, *t*_16_ = 1.51, *P*-value = 0.15).

**Fig 7:**
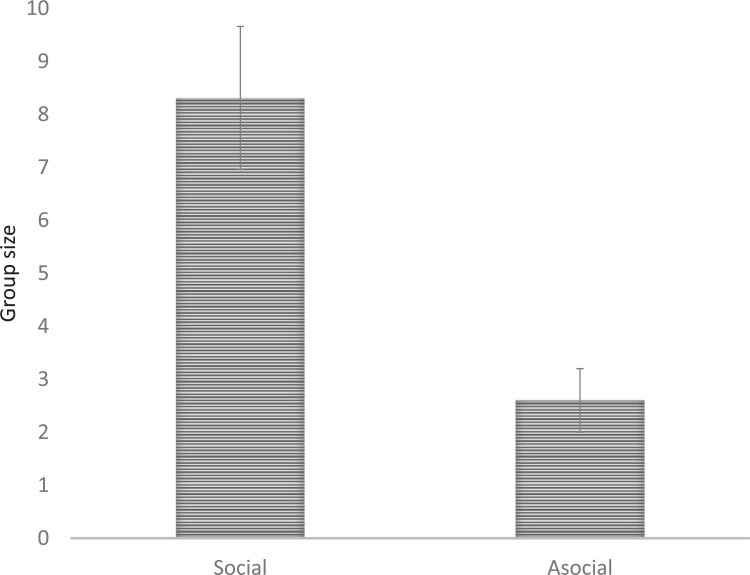
Comparison of the average group size between social and asocial episodes.

The present study also focused on a group size effect on the social interactions of finless porpoises because of the notions expressed in previous social experiences on the social associations that are developed among individuals. We compared the average number of aggregated individuals between gathering vs. non-gathering, rubbing vs. non-rubbing, and social vs. asocial episodes. A mean of 8.2 ± 1.7 aggregated individuals during five gathering episodes was not significantly different from a mean of 6.2 ± 1.5 individuals in 13 non-gathering episodes (*t* = 0.91, *df* = 10.18, *P*-value = 0.39). Similarly, the average number of aggregated individuals was not significantly different between the rubbing and non-rubbing episodes. A mean of 8.3 ± 1.8 individuals in 10 rubbing episodes was not significantly different from a mean of 4.8 ± 1.2 individuals in eight non-rubbing episodes (*t* = 1.68, *df* = 15.02, *P*-value = 0.11). However, a comparison between social and asocial episodes, which was represented by the combined presence or absence of the rubbing behavior and the gathering of individuals before and after the boats passed, showed a group size effect on social interaction (Fig 7). A mean of 8.3 ± 1.8 aggregated individuals in 13 social episodes was significantly different from a mean of 4.8 ± 1.2 individuals in five asocial episodes (*t* = 3.86, *df* = 15.40, *P*-value < 0.01). Finless porpoises showed a disperse tendency in one asocial episode of 5 individuals before a boat passed. During the rest of the asocial episodes, a pair of porpoises continued to swim together when the boat passed.

## Discussion

Even though the methods employed during this study had limitations, like an inability to identify a specific individual, our findings identified the social responses of wild finless porpoises to boat traffic around the Misumi West Port. Diving duration decreased significantly as a function of an increase in the number of aggregated individuals, suggesting a social response for mitigating an anthropogenic disturbance in a natural setting.

Boat traffic was a substantial disturbance to finless porpoises, and porpoises responded mostly by diving to avoid boats [25]. Minimizing the exposure time to a disturbance can benefit finless porpoises by allowing more energy to be allocated towards the primary behaviors associated with survival and reproduction rather than allocating energy and time to the avoidance behavior. Additionally, the travelling aggregations observed were in a non-feeding context and not a by-product of feeding. Thus, our results strongly support that finless porpoises respond socially to the risk of passing boats by aggregating; more individuals better mitigate the disturbance. Moreover, the aggregation size effect for suppressing the diving duration (Fig 6) does not necessarily implicate the development of long-lasting social bonds among the finless porpoises, as seen in social cetaceans (e.g., [14]). Rather, weak social relationships among the porpoises were indicated by group gathering and rubbing behaviors (Fig 7). This is not contradictory to the immediate benefits of the exchanges within the aggregations when boats pass, because a weakly bonded species may have difficulty in retaining the aggregation when there is no direct benefit from immediate concerns [43].

Nonetheless, our findings, using a new bird-eye’s observation technique, on the overall social tendency of finless porpoises with passing boats may generate new considerations on finless porpoise sociality. A drone enabled us to observe the behavior of finless porpoises, at an unprecedented resolution from a bird-eye angle, because of the contrast between the grey-white porpoise body color and the blue background of the seawater. As a result, we were able to successfully follow the aggregations of finless porpoises, with groups of up to 21 individuals, during each episode of boats passing. The fact that a solitary porpoise kept swimming at the seawater surface, without avoiding the passing boat, during four episodes ensured our data collection had no bias for aggregation episodes where solitary individuals kept extremely distant from the passing boats. Our results, free of the effects of data collection bias, raise additional quations on how group size influences the cohesion of finless porpoises. A mean of 6.7 ± 1.2 individuals within a group observed in our study is about three times larger than the group sizes reported in previous studies [25, 29]. The number of aggregated individuals during each episode, the group size effect on diving duration (Fig 6), and the social episodes (Fig 7) suggests the presence of social association in the aggregation to some extent, similar to the affiliative behaviors reported for groups of social animals [37]. Furthermore, the reaction times for diving and floating supports a tendency towards social cohesion among the aggregations; diving and floating reaction times were less than 20 s on average as described in a previous study [25], indicating that the porpoises, up to 21 individuals for an event, diving and floating was consistent with movement, space, and timing. In the large aggregations, the floating reaction movement tended to take more time to complete than in the smaller aggregations. However, the average reaction time was not different between diving and floating, which suggests that each individual kept the group floating cohesion consistent, even after the risk dissolved. This is comparable to the individuals that were diving under riskier conditions when the boats approached the aggregation. The tendency to disperse, manifested in a decrease in the number of individuals within an aggregation after a boat passed, only occurred in three of the episodes (out of 18 aggregated episodes) and indicates the potential development of social associations among finless porpoises beyond any forms of dyads, such as mother-calf [25].

The topographic characteristics around the Misumi West Port area was also expected to influence a relatively large number of the aggregated individuals in this study. The Misumi West Port is a narrow bay that connects the Ariake and Yatsushiro Sounds. The distances between individuals become forcibly closer when they travel in the bay. Considering the social tolerance tendency of finless porpoises in aggregations [29], several small groups of porpoises might aggregate as boats pass to share in the decreased risk to boat traffic. However, the premise that finless porpoises have flexible nesting social structures when there is relatively high individual density, implies, again, that there is a more complex social structure beyond the sociality associated with dyad forms. Our findings were limited for examining the social bonds among individuals, because of the reduced ability to identify specific individuals. Additional studies that focus on group size dynamics under a variety of conditions, using a technique that can discriminate between individuals, will be necessary to uncover the social structure of finless porpoises.

Finally, the present study showed the high potential of using drone-based observations to provide new insights on the current understanding of cetacean social and brain evolution. Ref. [28] argued the coevolution of the brain, social structure, and behavioral richness based on the social brain hypothesis [44], and the finless porpoise was characterized by small group sizes (a mean of 1.97 individuals) but relatively large brain sizes (3.71 encephalization quotient). A drone observation approach will offer new insights to finless porpoises and other cetacean behavior for things like behavioral repertoire richness, sociality, problem-solving tactics, and the similarities and differences among conspecific groups. Narrow-ridged finless porpoises are an endangered species with a risk of extinction and are only distributed in shallow coastal waters around the western Pacific Ocean from the Taiwan Strait to China, Korea, and Japan [45,46]. Finless porpoises in Japan are strictly controlled under the Act on the protection of fishery resources [47], while various anthropogenic activities, including bycatch from the fisheries and boat strikes, currently threaten their survival [48]. Our findings show that finless porpoises have some flexibility to the anthropogenic impacts they experience daily. However, considering all human impacts on marine ecosystems [3], we need to significantly reduce, at least, the direct impacts on finless porpoises and other endangered marine mammals. Our new observation technique using a drone enabled us to visualize the spatio-temporal overlapping of activity between finless porpoises and passing boats. The survery applied to a broad sea area will help in finding a better solution for mitigating the human-animal conflict involving porpoises and boats by segregating the spatio-temporal peak of activity for comprehesive marine mammal conservation. The protection of finless porpoise, and non-primate mammals, is undoubtedly crucial in understanding the evolutionary diversity of intelligence and sociality, which provides insight into the evolution of human intelligence and sociality (e.g., [49, 50]).

## Supporting information

S1 File Dataset. (XLSX)
